# Donor’s ability to comprehend and carefully assess the benefits and risks of donation in living donor liver transplantation: a Pakistani perspective – a short communication

**DOI:** 10.1097/MS9.0000000000001121

**Published:** 2023-07-31

**Authors:** Kaleem Ullah, Abdul W. Dogar, Sidhant Ochani, Hafiz B. Ahmad, Md. Al Hasibuzzaman

**Affiliations:** a Department of Liver Transplantation, Pir Abdul Qadir Shah Jeelani Institute of Medical Sciences, Gambat; b Department of Medicine, Khairpur Medical College, Khairpur Mir’s, Pakistan; c Institute of Nutrition and Food Science, University of Dhaka, Dhaka, Bangladesh

**Keywords:** donor knowledge, illiteracy-related comprehension, living liver donors, Pakistan

## Abstract

Patients unlikely to obtain deceased donor liver transplantation (DDLT) are offered living donor liver transplantation (LDLT) as an alternative. The success of LDLT is bound to the availability of altruistic donors who undergo smooth and safe surgery. Donor morbidity is reported to be up to 20–30%, while donor mortality is only 0.1–0.5%. Globally, LDLT poses numerous ethical concerns regarding living donors, such as autonomy, non-maleficence, and beneficence. The donor’s comprehension of information is a serious issue in LDLT. The donors may underestimate the risk of morbidity and mortality, as well as can ignore the long-term psychological consequences. Furthermore, donor voluntariness may be questionable as the donors may agree to donate under severe family pressure or emotional attachment. We propose open communication with all the donors, ensuring that they should not be subjected to any undue pressure or emotional lability. Donor knowledge and understanding of potential complications and the psychosocial aspect can be augmented by good communication. We also suggest that the donors’ education and psychological evaluation should be done in a friendly environment with complete privacy. Interventions should be aimed at improving communication and independent decision-making with the use of e-health educational tools for comprehension assessment.

*Dear Editor,*

Patients who are unlikely to obtain deceased donor liver transplantation (DDLT) are offered living donor liver transplantation (LDLT) as an alternative^1^. In Pakistan, DDLT has not yet been established due to multiple reasons, and only LDLT is actively practiced. The success of LDLT is bound to the availability of altruistic donors who undergo smooth and safe surgery. Donor morbidity is reported to be 10–20%, while donor mortality is only 0.1–0.5%^2^. In Pakistan, the Human Organ Transplant Authority (HOTA) regulates all transplant-related activities, and the Human Organ Transplantation Act 2010 is the main legislation that governs organ transplantation in Pakistan. This law regulates all aspects of organ transplantation, from the donation and retrieval of organs and postoperative care of patients. In the case of LDLT, HOTA prefers blood-related donations. The HOTA recommends that every potential organ donor will undergo a thorough medical evaluation to ensure that they are healthy enough to donate. Also, every transplant center in the country has its ethical committee that assesses the ethical, social, and legal aspects of donation. Institutional Ethics Committees adhere to the ethical guidelines as per the Pakistan National Guidelines for Human Research (2006), the Helsinki Declaration (2000), the CIOMS guidelines (2002), as well as the WHO publication on ‘Genomics and Global Health’ (2003). The Ethics Committee aims to guide the policies and practices related to organ donation and transplantation so that they are consistent with ethical principles. The transplant ethical committee also reviews the transplant evaluation process and confirms the relationship between the potential donor and recipient by verifying their national database record to ensure that no illegal donation takes place^1^.

Globally, LDLT poses numerous ethical concerns, such as autonomy, beneficence, and non-maleficence. The donor’s comprehension of the information regarding LDLT is a serious issue. That is why the donors may not only underestimate the risk of morbidity and mortality but also they can ignore the long-term psychological consequences of liver donation. Furthermore, donor voluntariness may be questionable as the donors may agree to donate under family pressure or emotional attachment^3^.

Health literacy is defined as a person’s ability to acquire, comprehend, communicate, and effectively participate in health-related discussions before making health decisions. Low health literacy is associated with poor decision-making^4^. The major contributing factors to health illiteracy are poverty, poor mental status, and a sub-standard education system^5^. According to the Pakistan Bureau of Statistics, one-third of the country’s population has got primary-level education only, and also women receive less education than men^6^. United Nations Educational, Scientific and Cultural Organization (UNESCO) ranked Pakistan 113 out of 120 in the Education development index^7^. Similarly, a local study on health literacy also reported that more than half of the study participants were labeled health illiterate^5^.

Health literacy can affect the donor’s ability to make an independent decision. Some potential donors may agree to donation, and others may forbid donation without weighing the actual risks and implications of donation due to a lack of knowledge and limited information regarding the donation process. For the transplant team, it becomes extremely difficult to make sure that the donor is autonomous, especially when the recipient is a close relative of the potential donor. The decision to donate an organ is influenced primarily by the recipient’s health concern rather than by the careful weighing of donor-related risks^3^.

Various interventions can be done to improve the health literacy of donors, like the provision of a health literacy booklet which should include images and content related to the process of donation, transplant procedure, and associated complications.

Launching public awareness campaigns can educate individuals about the organ donation process. These campaigns can include media outreach, educational programs in workplaces, and collaboration with various community social organizations. It is also possible to effectively capitalize on Pakistan’s recent massive telecommunications and social media booms to improve the health literacy of potential donors. Moreover, digital health literacy intervention can be introduced in the form of video tutorials having voice-overs in the local languages, and the narrative should be evident through images and symbols. And, even without voice-over, the audience will be able to understand the messages being communicated.

Focused and strategic partnerships between the health community and media seem to be the need of the hour. This would enable the media to be more socially accountable and will also help the transplant community to have more informed and knowledgeable potential donors with a better understanding. Sharing success stories of transplant patients on media-led health awareness campaigns can motivate more and more potential donors for organ donation. Transplant-related associations and societies should actively participate in improving the health literacy of potential donors. The role of the government is also critical in harnessing such programs. Government-led health programs need not only focus on health infrastructure, resources, and delivery but should also launch programs to improve the health literacy of potential donors.

Similarly, transplant clinicians in Pakistan also need to educate potential donors. Multiple guiding sessions should be held with these potential donors. Also, the way clinicians communicate must be individualized to each donor, keeping in view the country’s high health illiteracy which masks the donors’ ability to comprehend and carefully assess the benefits of donation against its risks. As caregivers, it is the responsibility of transplant clinicians to educate the donor in an understandable language during our consultations. This seemingly small action may have profound implications for making an autonomous decision.

We propose open communication with all the donors, ensuring that they should not be subjected to any pressure or emotional lability. Donors’ knowledge and understanding of potential complications and the psychosocial aspect can be augmented by good communication. We also suggest that the donors’ education and psychological evaluation should be done in a friendly environment with complete privacy. Furthermore, in elective circumstances, potential donors should be provided sufficient time to decide on donation before advancing with the donation process. Interventions should aim at improving communication and independent decision-making with the use of e-health educational tools. Major stakeholders, health authorities, social organizations, and media groups should capitalize on the available resources to create a meaningful impact and to improve the awareness and health literacy of potential liver donors.

All these interventions can smoothen the donation process and aid potential donors in deciding on liver donation.

## Ethical approval

Ethics approval was not required for this short communication.

## Consent

Informed consent was not required for this short communication.

## Sources of funding

None.

## Author contribution

K.U.: conceptualizing, manuscript writing, and review editing; A.W.D.: project administrator and review editing; S.O.: literature review, manuscript writing, referencing, and formatting; H.B.A.: literature review and manuscript writing; M.A.H.: review editing and formatting.

## Conflicts of interest disclosure

The authors declare that they have no conflicts of interest.

## Research registration unique identifying number (UIN)

None.

## Guarantor

All authors accept full responsibility for the work and/or the conduct of the study, had access to the data, and controlled the decision to publish.

## Data availability statement

Data sharing is not applicable to this article.

## Provenance and peer review

Not commissioned, externally peer-reviewed.
